# Supplementary material for: The brain structure, inflammatory, and genetic mechanisms mediate the association between physical frailty and depression
Source: Nat Commun. 2024 May 23;15:4411. doi: 10.1038/s41467-024-48827-8 (PMC11116547; doi:10.1038/s41467-024-48827-8)
Supplement: Supplementary file 1 — Supplementary Information [file 41467_2024_48827_MOESM1_ESM.pdf]

Supplementary Information

The brain structure, inflammatory, and genetic mechanisms mediate the association between physical frailty and depression

Rongtao Jiang[1], Stephanie Noble[2,3,4], Matthew Rosenblatt[5], Wei Dai[6], Jean Ye[7], Shu Liu[8,9], Shile Qi[10], Vince D. Calhoun[10], Jing Sui[10,11], Dustin Scheinost[1,5,7,12,13]

[1] Department of Radiology and Biomedical Imaging, Yale School of Medicine, New Haven, CT 06510, USA
[2] Department of Psychology, Northeastern University, Boston, MA, USA
[3] Department of Bioengineering, Northeastern University, Boston, MA, USA
[4] Center for Cognitive and Brain Health, Northeastern University, Boston, MA, USA
[5] Department of Biomedical Engineering, Yale University, New Haven, CT 06520, USA
[6] Department of Biostatistics, Yale University, New Haven, CT 06520, USA
[7] Interdepartmental Neuroscience Program, Yale University, New Haven, CT 06520, USA
[8] Department of Psychiatry, Amsterdam UMC, University of Amsterdam, Amsterdam, the Netherlands
[9] Amsterdam Neuroscience, Amsterdam, the Netherlands
[10] Tri-institutional Center for Translational Research in Neuroimaging and Data Science (TReNDS), Georgia State University, Georgia Institute of Technology, and Emory University, Atlanta, GA 30303, USA
[11] State Key Laboratory of Cognitive Neuroscience and Learning, Beijing Normal University, Beijing, China
[12] Department of Statistics & Data Science, Yale University, New Haven, CT 06520, USA
[13] Child Study Center, Yale School of Medicine, New Haven, CT 06510, USA

Supplementary table 1. Baseline characteristics by frailty status

	Whole population	Non-frail	Pre-frail	Frail
Total N	352277	202925	138111	11241
Age at baseline (mean, std)	56.48 (8.08)	56 (8.07)	57.04 (8.07)	58.34 (7.54)
Sex				
Females	182385 (51.77%)	99582 (49.07%)	75953 (54.99%)	6850 (60.94%)
Males	169892 (48.23%)	103343 (50.93%)	62158 (45.01%)	4391 (39.06%)
Race				
Ethnic minorities	17085 (4.85%)	6893 (3.4%)	8937 (6.47%)	1255 (11.16%)
White	335192 (95.15%)	196032 (96.6%)	129174 (93.53%)	9986 (88.84%)
Deprivation				
Higher	109352 (31.04%)	55140 (27.17%)	48331 (34.99%)	5881 (52.32%)
Middle	119860 (34.02%)	70628 (34.8%)	46107 (33.38%)	3125 (27.8%)
Lower	123065 (34.93%)	77157 (38.02%)	43673 (31.62%)	2235 (19.88%)
Family average Income				
Unknown	33208 (9.43%)	17959 (8.85%)	13931 (10.09%)	1318 (11.72%)
Low (<£51999)	231900 (65.83%)	126327 (62.25%)	96537 (69.9%)	9036 (80.38%)
Middle (£52000-£100k)	68532 (19.45%)	45570 (22.46%)	22204 (16.08%)	758 (6.74%)
High (>£100k)	18637 (5.29%)	13069 (6.44%)	5439 (3.94%)	129 (1.15%)
Education				
Less than college	231491 (65.71%)	125608 (61.9%)	96640 (69.97%)	9243 (82.23%)
Above College	120786 (34.29%)	77317 (38.1%)	41471 (30.03%)	1998 (17.77%)
Smoking status				
Never	194456 (55.2%)	115174 (56.76%)	74068 (53.63%)	5214 (46.38%)
Ever	157821 (44.8%)	87751 (43.24%)	64043 (46.37%)	6027 (53.62%)
Alcohol intake frequency				
Daily or almost daily	74925 (21.27%)	48092 (23.7%)	25553 (18.5%)	1280 (11.39%)
3-4 times a week	85542 (24.28%)	54744 (26.98%)	29474 (21.34%)	1324 (11.78%)
1-2 times a week	92219 (26.18%)	53089 (26.16%)	36615 (26.51%)	2515 (22.37%)
1-3 times a month	38818 (11.02%)	20509 (10.11%)	16853 (12.2%)	1456 (12.95%)
Special occasions only	36736 (10.43%)	16404 (8.08%)	17790 (12.88%)	2542 (22.61%)
Never	24037 (6.82%)	10087 (4.97%)	11826 (8.56%)	2124 (18.9%)
Sedentary behavior				
0-4 hours/day	254479 (72.24%)	155913 (76.83%)	93232 (67.51%)	5334 (47.45%)
>4 hours/day	97798 (27.76%)	47012 (23.17%)	44879 (32.49%)	5907 (52.55%)
Metabolic syndrome				
No	255464 (72.52%)	158211 (77.97%)	92046 (66.65%)	5207 (46.32%)
Yes	96813 (27.48%)	44714 (22.03%)	46065 (33.35%)	6034 (53.68%)
CRP (mean, std)	2.51 (4.22)	2.12 (3.68)	2.87 (4.53)	5.07 (7.19)

Quantitative variables were presented as mean with SD and categorical variables were presented as frequencies and percentages.

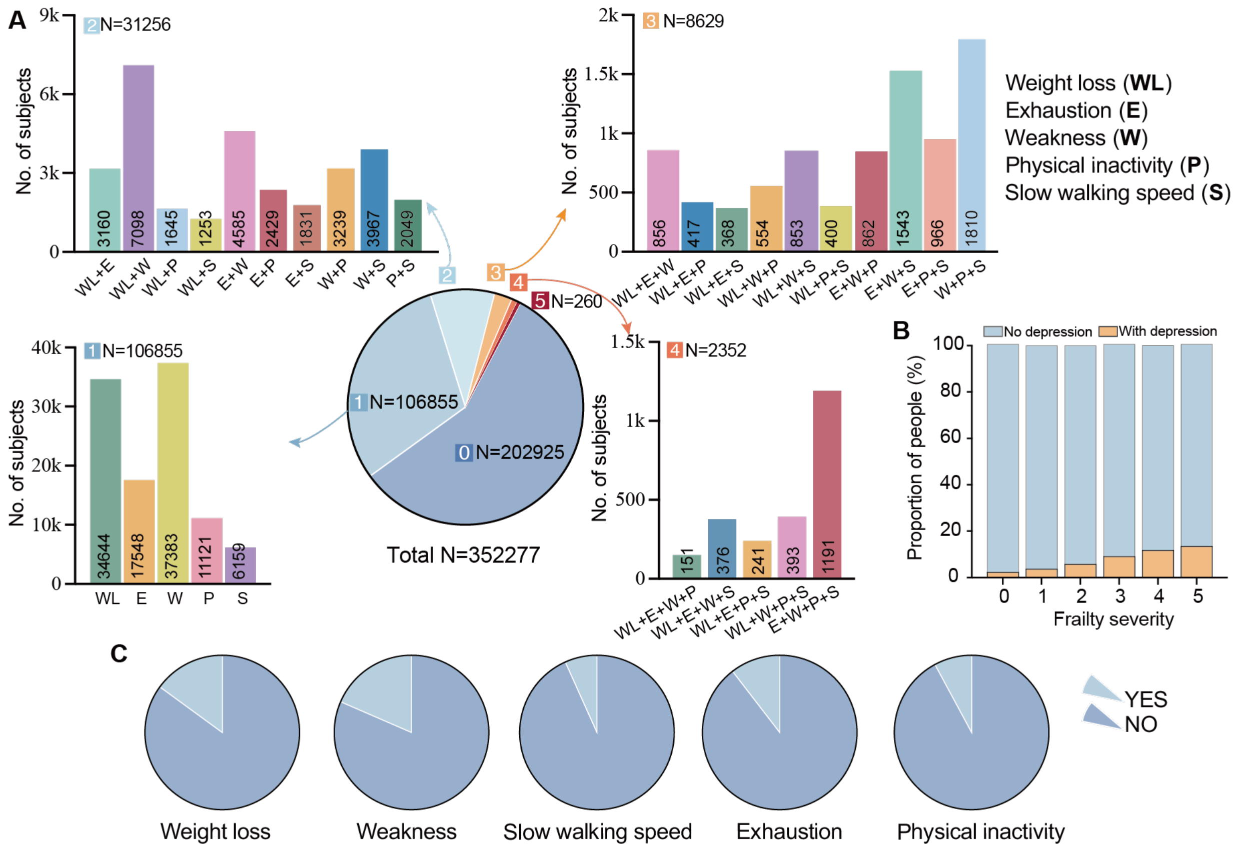

Supplementary Figure 1. (**A**) The number of participants with 0, 1, 2, 3, 4, and 5 frailty indicators was N=202925, N=106855, N=31256, N=8629, N=2352, and N=260, respectively. The barplot shows the number of subjects with different combinations of frailty indicators. (**B**) Prevalence of depression categorized by the number of frailty indicators. (**C**) The percentage of participants meeting the criterion for each frailty component.

Supplementary table 2. Associations between frailty status, each covariate and incident depression

	Participants	Events	HR (95% CI)	P value*
Frailty status				
Non-frail	202925	4595	1 (Ref)	
Prefrail	138111	5586	1.60 (1.53-1.66)	2.42×10^{-116}
Frail	11241	1088	3.20 (2.98-3.43)	6.25×10^{-231}
Age group				
Middle age	286135	9042	1 (Ref)	
Older age	66142	2227	0.92 (0.87-0.96)	3.17×10^{-4}
Sex				
Females	182385	6818	1 (Ref)	
Males	169892	4451	0.73 (0.71-0.76)	6.02×10^{-55}
Race				
Ethnic minorities	17085	489	1 (Ref)	
White	335192	10780	1.41 (1.29-1.55)	4.66×10^{-13}
Deprivation				
Higher	109352	4450	1 (Ref)	
Middle	119860	3592	0.83 (0.80-0.87)	6.98×10^{-16}
Lower	123065	3227	0.78 (0.74-0.82)	1.98×10^{-25}
Family average Income				
Unknown	33208	1056	1 (Ref)	
Low (<£51999)	231900	8447	1.15 (1.07-1.22)	3.28×10^{-5}
Middle (£52000-£100k)	68532	1515	0.89 (0.82-0.97)	6.59×10^{-3}
High (>£100k)	18637	251	0.59 (0.51-0.68)	9.92×10^{-14}
Education				
Less than college	231491	8295	1 (Ref)	
Above College	120786	2974	0.89 (0.85-0.93)	1.40×10^{-7}
Smoking status				
Never	194456	5285	1 (Ref)	
Ever	157821	5984	1.34 (1.29-1.39)	1.21×10^{-50}
Alcohol intake frequency				
Daily or almost daily	74925	2063	1 (Ref)	
3-4 times a week	85542	2205	0.94 (0.89-1)	0.062
1-2 times a week	92219	2794	1.01 (0.95-1.07)	0.827
1-3 times a month	38818	1444	1.14 (1.07-1.22)	1.67×10^{-4}
Special occasions only	36736	1628	1.21 (1.13-1.29)	4.68×10^{-8}
Never	24037	1135	1.33 (1.23-1.44)	1.67×10^{-13}
Sedentary behavior				
0-4 hours/day	254479	7297	1 (Ref)	
>4 hours/day	97798	3972	1.10 (1.05-1.14)	6.54×10^{-6}
Metabolic syndrome				
No	255464	7273	1 (Ref)	
Yes	96813	3996	1.18 (1.13-1.23)	1.85×10^{-15}

*All P values were two-sided, and no adjustments were made for multiple comparisons.

Frailty	Total N	Cases	HR (95% CI)	P value
**Frailty status**				
Nonfrail	198716	902	Reference	
Prefrail	133230	1051	1.54 (1.41−1.69)	1.01×10^{-20}
Frail	10302	176	2.68 (2.26−3.17)	9.45×10^{-30}
**Frailty severity**				
0	198716	902	Reference	
1	103503	724	1.41 (1.28−1.56)	7.20×10^{-12}
2	29727	327	2.00 (1.75−2.28)	4.55×10^{-25}
3	7960	131	2.68 (2.21−3.24)	3.39×10^{-24}
≥4	2342	45	2.88 (2.12−3.91)	1.26×10^{-11}

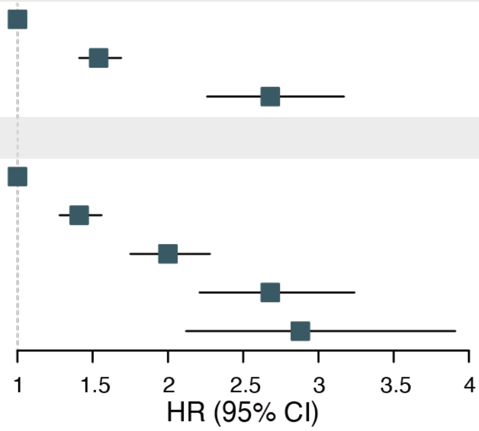

Supplementary Figure 2. Associations of frailty status and frailty severity with depression incidence when using a 10-year landmark analysis, where participants who experienced events within the first 10 years of follow-up were excluded. This analysis included 342248 participants, of whom 10302 met the criteria for frailty, 133230 for prefrailty, and 198716 for non-frailty. We collapsed participants with four or five indicators of frailty due to a limited number of depression cases in those fulfilling five indicators. Dots: mean HRs; Horizontal lines: 95% CI. All P values were two-sided, and no adjustments were made for multiple comparisons. Source data are provided as a Source Data file.

Supplementary table 3. Associations between frailty status and incident depression when including an extended set of covariates

	Participants	Events	HR (95% CI)	P value*
Frailty status				
Non-frail	182772	4234	1 (Ref)	
Prefrail	126212	5255	1.57 (1.51-1.64)	2.29×10^{-101}
Frail	10152	1019	2.98 (2.77-3.21)	2.53×10^{-185}
Age group				
Middle age	258912	8396	1 (Ref)	
Older age	60224	2112	0.91 (0.86-0.96)	3.65×10^{-4}
Sex				
Females	164679	6368	1 (Ref)	
Males	154457	4140	0.73 (0.70-0.76)	3.64×10^{-54}
Race				
Ethnic minorities	15820	444	1 (Ref)	
White	303316	10064	1.53 (1.39-1.69)	3.54×10^{-17}
Deprivation				
Higher	97026	4086	1 (Ref)	
Middle	110046	3369	0.84 (0.80-0.88)	2.32×10^{-12}
Lower	112064	3053	0.81 (0.77-0.85)	1.67×10^{-15}
Family average Income				
Unknown	29563	964	1 (Ref)	
Low (<£51999)	210785	7909	1.15 (1.07-1.23)	6.40×10^{-5}
Middle (£52000-£100k)	61983	1406	0.92 (0.84-1.00)	0.0494
High (>£100k)	16805	229	0.59 (0.51-0.68)	1.71×10^{-12}
Education				
Less than college	211705	7794	1 (Ref)	
Above College	107431	2714	0.89 (0.85-0.93)	4.37×10^{-7}
Smoking status				
Never	176059	4955	1 (Ref)	
Ever	143077	5553	1.32 (1.27-1.37)	6.9×10^{-42}
Alcohol intake frequency				
Daily or almost daily	68444	1946	1 (Ref)	
3-4 times a week	77514	2035	0.93 (0.88-0.99)	0.0315
1-2 times a week	83261	2607	1.00 (0.95-1.07)	0.904
1-3 times a month	35130	1360	1.14 (1.06-1.22)	3.68×10^{-4}
Special occasions only	33214	1508	1.17 (1.09-1.26)	8.20×10^{-6}
Never	21573	1052	1.3 (1.2-1.4)	1.11×10^{-10}
Sedentary behavior				
0-4 hours/day	230365	6806	1 (Ref)	
>4 hours/day	88771	3702	1.07 (1.03-1.12)	1.09×10^{-3}
Metabolic syndrome				
No	229967	6711	1 (Ref)	
Yes	89169	3797	1.18 (1.13-1.23)	7.56×10^{-15}
Sleep				

7-9 h/day	237832	6961	1 (Ref)	
>9 h/day	4274	304	1.66 (1.48-1.86)	1.69×10^{-17}
<7 h/day	77030	3243	1.28 (1.23-1.33)	4.72×10^{-30}
Cancer diagnosis				
No	295142	9559	1 (Ref)	
Yes	23994	949	1.07 (1.00-1.15)	0.0361
Household size				
One person	55424	2425	1 (Ref)	
Two people	151955	4765	0.85 (0.81-0.89)	1.53×10^{-10}
>=3 people	111757	3318	0.93 (0.88-0.99)	0.0173
Employment status				
Not employed	126349	4849	1 (Ref)	
Employed	192787	5659	0.92 (0.87-0.96)	1.43×10^{-4}
Air pollution				
Low	110438	3221	1 (Ref)	
Middle	105013	3378	0.99 (0.95-1.04)	0.804
High	103685	3909	1.05 (1.00-1.11)	0.0493

*All P values were two-sided, and no adjustments were made for multiple comparisons.

Supplementary table 4. Associations between physical frailty and incident depression by age and sex

	Pre-frailty		Frailty	
	HR (95% CI)	P for interaction*	HR (95% CI)	P for interaction*
Males	1.52 (1.44-1.60)	3.60×10^{-2}	2.90 (2.65-3.17)	3.76×10^{-3}
Females	1.69 (1.59-1.80)		3.66 (3.27-4.10)	
Age≤65 years	1.66 (1.59-1.74)	6.08×10^{-6}	3.37 (3.10-3.66)	1.62×10^{-3}
Age>65 years	1.30 (1.19-1.43)		2.53 (2.18-2.93)	

*All P values were two-sided, and no adjustments were made for multiple comparisons.

Supplementary table 5. Associations of frailty and its components with incident depression

	Independent association		Mutually adjusted association		
	**HR (95% CI)**	**P value**	**HR (95% CI)**	**P value**	**PAF**
Weight loss	1.28 (1.22-1.34)	2.15×10^{-24}	1.28 (1.22-1.34)	7.00×10^{-24}	4.21%
Exhaustion	2.29 (2.19-2.40)	2.20×10^{-286}	2.10 (2.00-2.20)	2.40×10^{-217}	13.07%
Weakness	1.36 (1.31-1.42)	7.50×10^{-44}	1.23 (1.18-1.29)	3.55×10^{-19}	7.11%
Physical inactivity	1.43 (1.35-1.51)	2.90×10^{-35}	1.15 (1.08-1.22)	1.30×10^{-05}	4.04%
Slow walking speed	1.91 (1.80-2.01)	8.50×10^{-118}	1.50 (1.42-1.59)	4.65×10^{-42}	7.07%
Prefrail	1.60 (1.53-1.66)	2.42×10^{-116}			20.58%
Frail	3.20 (2.98-3.43)	6.25×10^{-231}			13.16%

The independent association between components of physical frailty and depression incidence was examined by including only one component of frailty as an exposure variable in the Cox proportional hazard model. The mutually adjusted association between components of frailty and depression incidence was examined by including all five components simultaneously in the Cox proportional hazard model[14]. Age, sex, race, alcohol intake, smoking status, sedentary behavior, education level, material deprivation, family income, and metabolic syndrome were treated as confounders being associated with both exposures and outcomes in all models. PAF, population attributable fraction. All P values were two-sided, and no adjustments were made for multiple comparisons.

Supplementary table 6. Characteristic of the 30 physical frailty associated SNPs

SNP	Effect allele	Other allele	Beta	SE	P value*
rs1048027	T	C	-0.011	0.0019	3.80E-08
rs10828258	A	G	-0.014	0.002	7.50E-13
rs11130207	T	G	0.015	0.0025	5.30E-10
rs11150602	A	G	0.011	0.0019	2.00E-08
rs11660938	G	T	-0.011	0.0019	1.50E-08
rs11689546	A	G	-0.012	0.0019	2.90E-10
rs12601919	A	G	-0.013	0.0023	3.20E-08
rs12712072	A	G	0.015	0.0022	1.60E-11
rs13107325	C	T	-0.031	0.0035	7.60E-19
rs1421085	T	C	-0.013	0.0019	3.00E-12
rs17707300	T	C	-0.012	0.0019	2.50E-10
rs17716502	C	T	0.013	0.0023	9.00E-09
rs2044169	A	G	-0.011	0.002	3.90E-08
rs2287234	C	A	0.011	0.0018	1.10E-08
rs2847308	C	T	0.012	0.0019	5.90E-10
rs28509789	T	G	0.012	0.0021	3.30E-08
rs303762	T	C	-0.011	0.0019	3.80E-08
rs362307	C	T	-0.029	0.0035	1.50E-16
rs3821269	A	G	0.011	0.0018	2.50E-09
rs4457304	A	C	0.01	0.0018	2.00E-08
rs4549685	C	T	0.011	0.0019	1.80E-08
rs62082234	T	G	-0.011	0.0019	4.10E-08
rs62444907	C	T	0.019	0.0025	3.10E-13
rs6751993	A	G	-0.015	0.0025	2.50E-09
rs724701	A	G	0.012	0.0021	1.00E-08
rs72709800	G	A	-0.021	0.0034	8.80E-10
rs7703746	G	A	0.013	0.0018	6.40E-13
rs80032406	T	C	-0.013	0.0021	1.30E-09
rs8044920	C	T	-0.011	0.0019	1.60E-08
rs9953231	G	A	-0.012	0.0022	3.00E-08

*All P values were two-sided, and no adjustments were made for multiple comparisons.

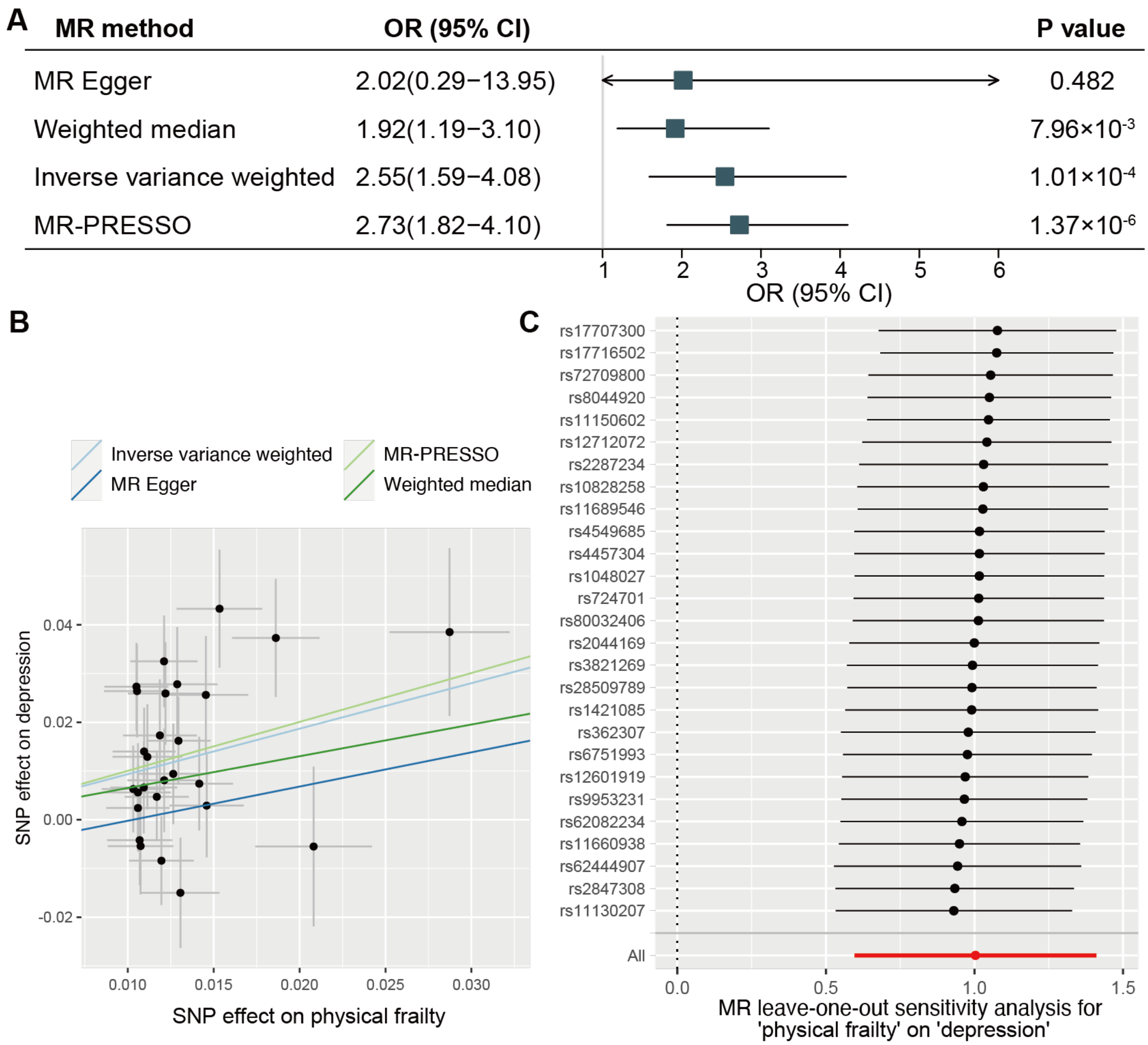

Supplementary Figure 3. Mendelian randomization plots for physical frailty ->risk of depression with SNPs selected under the threshold of 5.0×10^{-8}. (A) The causal relationship between physical frailty and depression based on Mendelian randomization analyses. Dots: mean ORs; Horizontal lines: 95% CI. (B) Scatterplot of SNP effects on physical frailty versus their effects on depression, with the slope of each line representing estimated MR effect per method. (C) Forest plot of leave-one-SNP-out sensitivity analysis. The significant SNPs were selected under the threshold of 5.0×10^{-8}, and three outliers detected by MR-PRESSO were removed from the MR analysis. Dots: estimated beta coefficients; Horizontal lines: 95% CI. All P values were two-sided, and no adjustments were made for multiple comparisons. Source data are provided as a Source Data file.

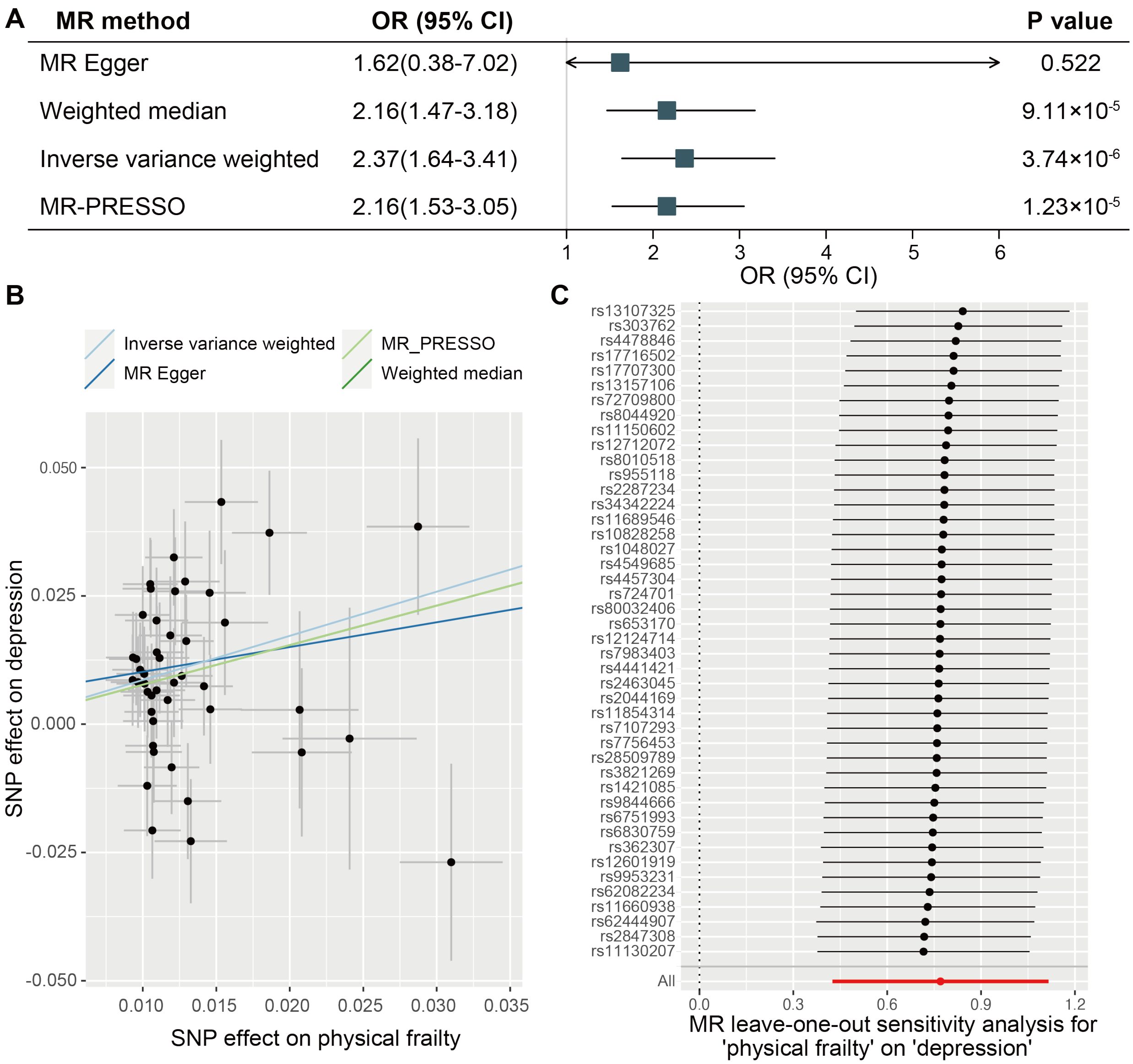

Supplementary Figure 4. Mendelian randomization plots for physical frailty ->risk of depression with SNPs selected under the threshold of 5.0×10^{-7}. (A) The causal relationship between physical frailty and depression based on Mendelian randomization analyses. Dots: mean ORs; Horizontal lines: 95% CI. (B) Scatterplot of SNP effects on physical frailty versus their effects on depression, with the slope of each line representing estimated MR effect per method. (C) Forest plot of leave-one-SNP-out sensitivity analysis. The significant SNPs were selected under a threshold of 5.0×10^{-7}, and one outlier detected by MR-PRESSO was removed from the MR analysis. Dots: estimated beta coefficients; Horizontal lines: 95% CI. All P values were two-sided, and no adjustments were made for multiple comparisons. Source data are provided as a Source Data file.

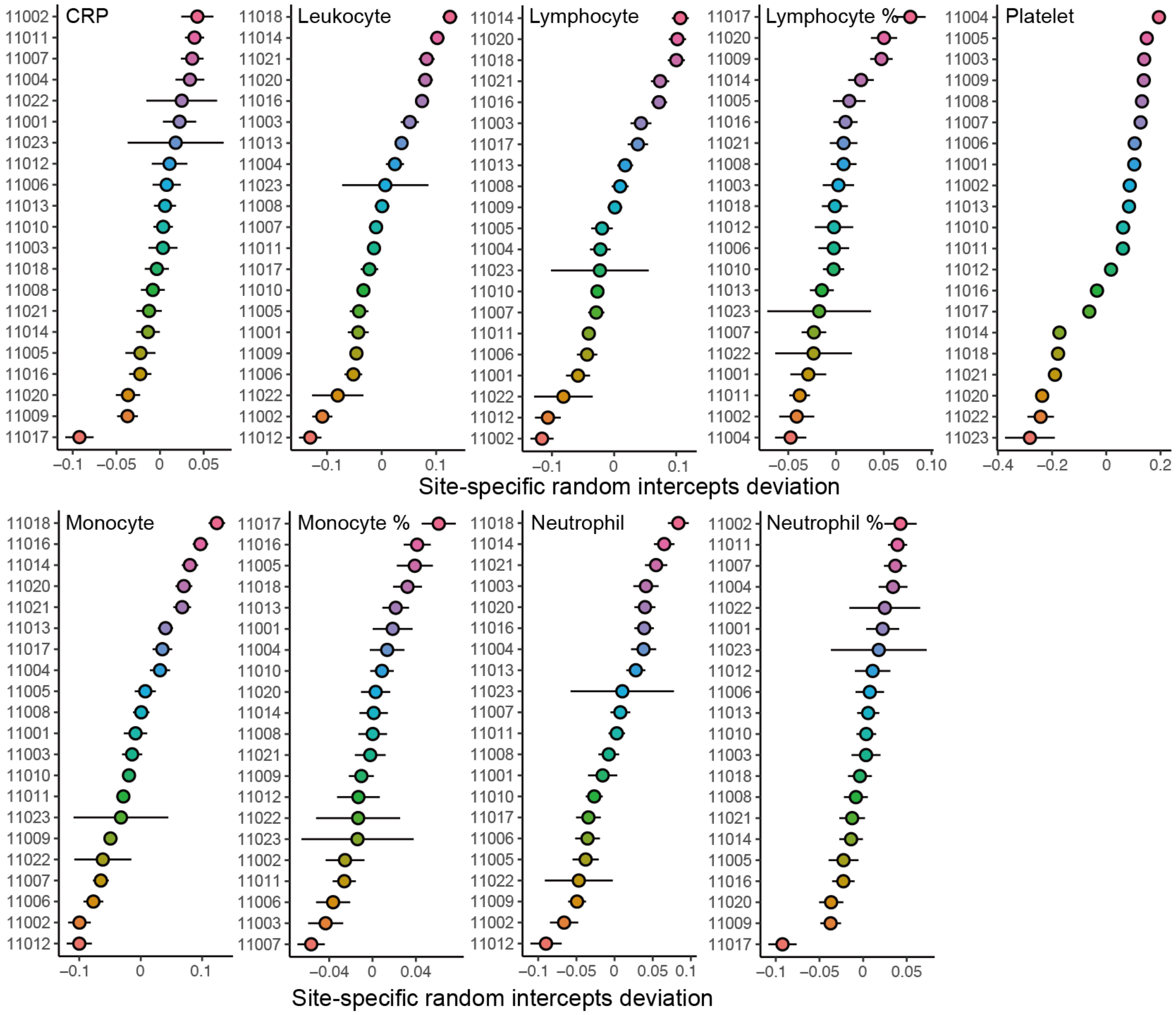

Supplementary Figure 5. The distribution of random coefficients for each UK Biobank assessment center. A linear-mixed effect model was applied to investigate the association of physical frailty with nine inflammatory markers. Within the same analytical framework, physical frailty was fitted as a fixed effect, the UK Biobank assessment center as a random effect, and each of the nine inflammatory markers was set as the dependent variable in separate models. The number of participants used for each inflammatory marker can be found at Table S7. Analysis employed linear mixed-effects models, which tested the statistical significance of coefficients against a t-distribution. Dots: estimated beta coefficients; Horizontal lines: 95% CI. Source data are provided as a Source Data file.

Supplementary table 7. Associations between frailty severity and inflammation markers

	Coeff	SE	P value*	Cohen's d	N
**CRP**	0.12	0.00166	$<10^{-300}$	0.24	352277
**Leukocyte**	0.06	0.00174	1.09×10^{-286}	0.12	342268
**Lymphocyte**	0.01	0.00176	1.39×10^{-15}	0.02	342771
**Lymphocyte%**	-0.04	0.00177	4.33×10^{-104}	-0.08	342775
**Platelet**	0.03	0.00173	1.28×10^{-51}	0.06	343371
**Monocyte**	0.03	0.00173	1.67×10^{-71}	0.06	342771
**Monocyte%**	-0.01	0.00174	7.88×10^{-15}	-0.02	342775
**Neutrophil**	0.07	0.00176	$<10^{-300}$	0.14	342771
**Neutrophil%**	0.04	0.00179	1.05×10^{-92}	0.08	342775

*All P values were two-sided, and no adjustments were made for multiple comparisons.

Supplementary table 8. Associations between inflammation markers and incident depression

	Linear association between inflammation and incident depression		Associations between inflammation tertile and incident depression			
			Middle tertile		Highest tertile	
	HR (95% CI)	P value*	HR (95% CI)	P value*	HR (95% CI)	P value*
**CRP**	1.10 (1.08-1.12)	1.24×10^{-21}	1.06 (1.01-1.12)	0.13	1.22 (1.16-1.28)	7.14×10^{-15}
**Leukocyte**	1.10 (1.08-1.12)	3.64×10^{-23}	1.12 (1.06-1.17)	1.03×10^{-4}	1.25 (1.19-1.31)	1.59×10^{-19}
**Lymphocyte**	1.03 (1.02-1.05)	3.06×10^{-3}	1.04 (0.99-1.09)	0.99	1.09 (1.04-1.14)	3.33×10^{-3}
**Lymphocyte%**	0.95 (0.93-0.97)	5.64×10^{-6}	0.88 (0.84-0.93)	1.49×10^{-6}	0.89 (0.85-0.93)	3.05×10^{-6}
**Platelet**	1.06 (1.04-1.08)	4.49×10^{-8}	1.00 (0.95-1.05)	0.936	1.11 (1.06-1.17)	7.86×10^{-5}
**Monocyte**	1.05 (1.03-1.07)	2.50×10^{-5}	1.07 (1.02-1.14)	0.13	1.10 (1.06-1.15)	5.17×10^{-5}
**Monocyte%**	0.97 (0.96-0.99)	0.10	0.97 (0.93-1.01)	0.99	0.97 (0.92-1.02)	0.99
**Neutrophil**	1.10 (1.08-1.12)	4.64×10^{-26}	1.06 (1.01-1.11)	0.18	1.25 (1.20-1.31)	4.29×10^{-20}
**Neutrophil%**	1.05 (1.03-1.07)	3.28×10^{-6}	1.02 (0.97-1.07)	0.99	1.11 (1.06-1.16)	1.25×10^{-4}

*All P values were two-sided, and no adjustments were made for multiple comparisons.

Supplementary table 9. Baseline characteristics of matched participants

	Whole population	Non-frail	Frail	P for difference*
Total N	22482	11241	11241	
Age group				0.926
Middle age	16945 (75.37%)	8469 (75.34%)	8476 (75.4%)	
Older age	5537 (24.63%)	2772 (24.66%)	2765 (24.6%)	
Sex				0.732
Females	13726 (61.05%)	6876 (61.17%)	6850 (60.94%)	
Males	8756 (38.95%)	4365 (38.83%)	4391 (39.06%)	
Race				0.37
Ethnic minorities	2467 (10.97%)	1212 (10.78%)	1255 (11.16%)	
White	20015 (89.03%)	10029 (89.22%)	9986 (88.84%)	
Deprivation				0.956
Higher	11754 (52.28%)	5873 (52.25%)	5881 (52.32%)	
Middle	6269 (27.88%)	3144 (27.97%)	3125 (27.8%)	
Lower	4459 (19.83%)	2224 (19.78%)	2235 (19.88%)	
Family average Income				0.782
Unknown	2621 (11.66%)	1303 (11.59%)	1318 (11.72%)	
Low (<£51999)	18113 (80.57%)	9077 (80.75%)	9036 (80.38%)	
Middle (£52000-£100k)	1504 (6.69%)	746 (6.64%)	758 (6.74%)	
High (>£100k)	244 (1.09%)	115 (1.02%)	129 (1.15%)	
Education qualifications				0.649
Less than college	18513 (82.35%)	9270 (82.47%)	9243 (82.23%)	
Above College	3969 (17.65%)	1971 (17.53%)	1998 (17.77%)	
Smoking				0.862
Never	10442 (46.45%)	5228 (46.51%)	5214 (46.38%)	
Ever	12040 (53.55%)	6013 (53.49%)	6027 (53.62%)	
Alcohol intake frequency				0.485
Daily or almost daily	2564 (11.4%)	1284 (11.42%)	1280 (11.39%)	
3-4 times a week	2644 (11.76%)	1320 (11.74%)	1324 (11.78%)	
1-2 times a week	5034 (22.39%)	2519 (22.41%)	2515 (22.37%)	
1-3 times a month	2941 (13.08%)	1485 (13.21%)	1456 (12.95%)	
Special occasions only	5162 (22.96%)	2620 (23.31%)	2542 (22.61%)	
Never	4137 (18.4%)	2013 (17.91%)	2124 (18.9%)	
Sedentary behavior				0.862
0-4 hours/day	10654 (47.39%)	5320 (47.33%)	5334 (47.45%)	
>4 hours/day	11828 (52.61%)	5921 (52.67%)	5907 (52.55%)	
Metabolic syndrome				0.504
No	10465 (46.55%)	5258 (46.78%)	5207 (46.32%)	
Yes	12017 (53.45%)	5983 (53.22%)	6034 (53.68%)	

* All P values were two-sided, and no adjustments were made for multiple comparisons.

Supplementary table 10. Associations between frailty and inflammation markers based on matched data

	Coeff	SE	P value*	Cohen's d	N
CRP	0.232	0.0065	3.73×10^{-272}	0.48	11241 *vs* 11241
Leukocyte	0.127	0.0067	7.88×10^{-79}	0.26	11241 vs 11241
Lymphocyte	0.016	0.0068	0.174	0.03	11241 vs 11241
Lymphocyte%	-0.091	0.0067	1.62×10^{-40}	-0.18	11241 vs 11241
Platelet	0.056	0.0068	7.59×10^{-16}	0.11	11241 vs 11241
Monocyte	0.064	0.0068	2.64×10^{-20}	0.13	11241 vs 11241
Monocyte%	-0.025	0.0068	2.18×10^{-3}	-0.05	11241 vs 11241
Neutrophil	0.146	0.0067	7.47×10^{-104}	0.3	11241 vs 11241
Neutrophil%	0.087	0.0067	5.54×10^{-37}	0.17	11241 vs 11241

* All P values were two-sided, and no adjustments were made for multiple comparisons.

Supplementary table 11. Results of the mediation analyses between frailty, incident depression, and inflammation markers using matched data

	Total No.	Prop. Mediated (%)	95% CI Lower-Upper		P value*
CRP	22482	3.18	1.03	5.41	0.0028
Leukocyte	21858	2.34	1.09	3.69	2.0×10^{-4}
Lymphocyte	21806	0.21	0.02	0.48	0.0244
Lymphocyte%	21806	0.33	-0.47	1.20	0.436
Platelet	21858	0.26	-0.30	0.80	0.346
Monocyte	21806	0.53	-0.03	1.15	0.064
Monocyte%	21806	0.11	-0.11	0.39	0.324
Neutrophil	21806	2.34	1.00	3.69	8.0×10^{-4}
Neutrophil%	21806	0.39	-0.42	1.20	0.319

* All P values were two-sided, and no adjustments were made for multiple comparisons.

Supplementary table 12. Brain regions showing significant associations with physical frailty

Brain region	Coeff	se	P_{FDR}*	T value	Cohen's d
R.heschl_gyrus	-0.035	0.0064	3.72×10^{-6}	-5.52	-0.07
R.parahipp_gyrus_ant	-0.034	0.0068	2.47×10^{-5}	-4.97	-0.068
R.insular_cortex	-0.031	0.0066	5.32×10^{-5}	-4.72	-0.062
R.postcent_gyrus	-0.031	0.0061	2.47×10^{-5}	-5.03	-0.062
L.parietal_operc_cortex	-0.031	0.0068	7.50×10^{-5}	-4.58	-0.062
L.temp_fusif_cortex_ant	-0.03	0.0067	9.99×10^{-5}	-4.47	-0.06
R.front_med_cortex	-0.029	0.0067	1.32×10^{-4}	-4.36	-0.058
R.temp_fusif_cortex_ant	-0.029	0.0068	1.32×10^{-4}	-4.34	-0.058
R.thalamus	-0.029	0.006	3.63×10^{-5}	-4.84	-0.058
L.ventral_striatum	-0.029	0.0064	9.99×10^{-5}	-4.46	-0.058
R.cent_operc_cortex	-0.028	0.0064	1.08×10^{-4}	-4.42	-0.056
L.thalamus	-0.028	0.006	5.79×10^{-5}	-4.66	-0.056
L.insular_cortex	-0.027	0.0065	2.53×10^{-4}	-4.16	-0.054
L.parahipp_gyrus_ant	-0.027	0.0068	2.56×10^{-4}	-3.93	-0.054
L.heschl_gyrus	-0.027	0.0065	2.23×10^{-4}	-4.2	-0.054
L.supramarg_gyrus_post	-0.026	0.0069	6.97×10^{-4}	-3.82	-0.052
R.pallidum	-0.026	0.0067	6.97×10^{-4}	-3.82	-0.052
R.hippocampus	-0.026	0.0065	3.34×10^{-4}	-4.08	-0.052
R.precentral_gyrus	-0.025	0.0063	5.18×10^{-4}	-3.95	-0.05
L.cent_operc_cortex	-0.025	0.0064	6.97×10^{-4}	-3.83	-0.05
R.ventral_striatum	-0.025	0.0063	4.49×10^{-4}	-3.99	-0.05
R.parietal_operc_cortex	-0.024	0.0068	2.20×10^{-3}	-3.5	-0.048
L.pallidum	-0.024	0.0068	2.24×10^{-3}	-3.47	-0.048
L.front_med_cortex	-0.023	0.0067	2.48×10^{-3}	-3.42	-0.046
R.juxtapos_lobule_cortex	-0.023	0.0067	2.24×10^{-3}	-3.47	-0.046
R.planum_temporale	-0.023	0.0067	2.20×10^{-3}	-3.49	-0.046
L.putamen	-0.023	0.0065	1.73×10^{-3}	-3.58	-0.046
R.temp_fusif_cortex_post	-0.022	0.0067	4.44×10^{-3}	-3.25	-0.044
R.putamen	-0.022	0.0065	2.32×10^{-3}	-3.45	-0.044
R.subcallosal_cortex	-0.02	0.0069	1.44×10^{-2}	-2.86	-0.04
L.planum_temporale	-0.02	0.0068	1.05×10^{-2}	-2.98	-0.04
R.front_operc_cortex	-0.019	0.0066	1.57×10^{-2}	-2.83	-0.038
L.hippocampus	-0.019	0.0064	1.36×10^{-2}	-2.89	-0.038
R.supramarg_gyrus_ant	-0.018	0.0068	2.04×10^{-2}	-2.7	-0.036
L.juxtapos_lobule_cortex	-0.018	0.0068	2.14×10^{-2}	-2.67	-0.036
L.subcallosal_cortex	-0.018	0.0067	1.91×10^{-2}	-2.74	-0.036
R.precun_cortex	-0.018	0.0064	1.59×10^{-2}	-2.81	-0.036
L.occ_fusif_gyrus	-0.018	0.0066	1.92×10^{-2}	-2.73	-0.036

L.latocc_cortex_sup	-0.017	0.0063	1.96×10^{-2}	-2.72	-0.034
L.precun_cortex	-0.017	0.0065	2.58×10^{-2}	-2.59	-0.034
R.occ_fusif_gyrus	-0.017	0.0066	2.47×10^{-2}	-2.61	-0.034
R.caudate	-0.017	0.0068	3.01×10^{-2}	-2.53	-0.034
R.sup_temp_gyrus_post	-0.016	0.0066	4.45×10^{-2}	-2.36	-0.032
R.front_orb_cortex	-0.015	0.0064	4.50×10^{-2}	-2.35	-0.03
L.planum_polare	-0.015	0.0063	3.70×10^{-2}	-2.44	-0.03
R.planum_polare	-0.015	0.0062	3.70×10^{-2}	-2.44	-0.03

*All P values were two-sided and corrected using the Benjamini-Hochberg false discovery rate (FDR) method.

Supplementary table 13. Brain regions showing significant associations with PHQ-9

Brain region	Coeff	se	P_{FDR}*	T value	Cohen's d
R.precentral_gyrus	-0.024	0.0063	6.60×10^{-3}	-3.76	-0.048
L.subcallosal_cortex	-0.023	0.0067	2.16×10^{-2}	-3.36	-0.046
L.thalamus	-0.023	0.0061	6.60×10^{-3}	-3.78	-0.046
R.thalamus	-0.023	0.0061	6.60×10^{-3}	-3.75	-0.046
R.ventral_striatum	-0.021	0.0063	2.22×10^{-2}	-3.29	-0.042
L.precentral_gyrus	-0.019	0.0063	3.96×10^{-2}	-3.02	-0.038
L.postcent_gyrus	-0.019	0.0062	3.96×10^{-2}	-3.02	-0.038

*All P values were two-sided and corrected using the Benjamini-Hochberg false discovery rate (FDR) method.

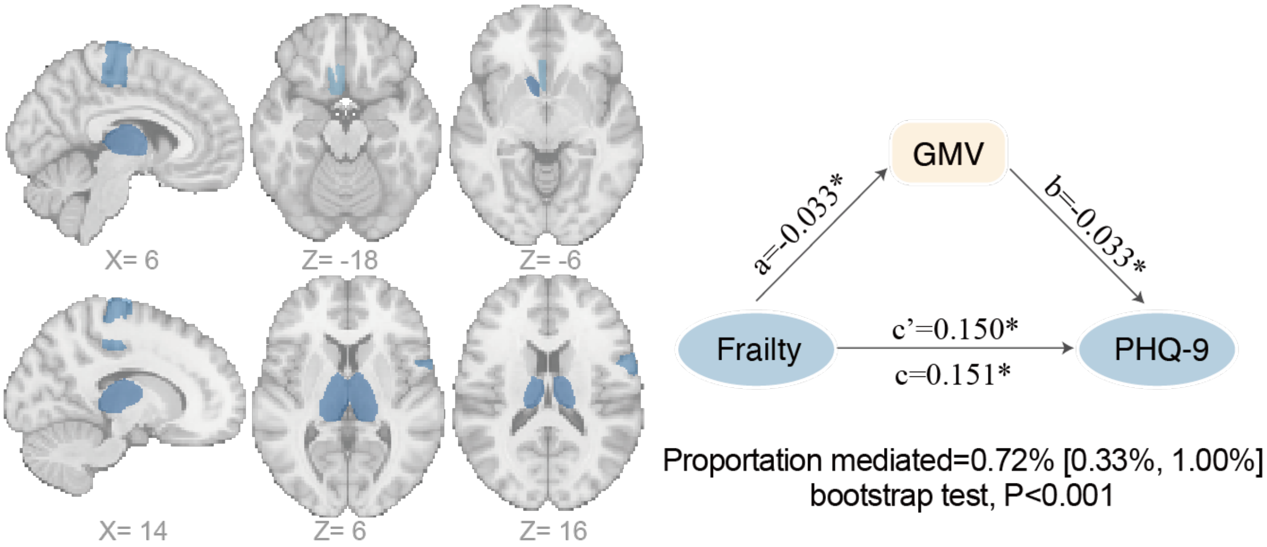

Supplementary Figure 6. Five brain regions showed consistent associations with both frailty and PHQ-9 scores, including the left and right thalamus, right precentral gyrus, left subcallosal cortex, and right ventral striatum. Further analysis indicated that the mean GMV of these five brain regions significantly mediated the association between physical frailty and PHQ-9 scores (two-sided, unadjusted P<0.001, 5000 bootstrapping test, proportion of mediated variance 0.72%, 95% CI=[0.33%, 1.00%], n=21,346). *two-sided P<0.05. Source data are provided as a Source Data file.

Supplementary table 14. Association of alcohol status and frailty status with depression

	Number of subjects (percentage)			Prospective association with depression	
**Alcohol status**	**Non-frail**	**Pre-frail**	**Frail**	**HR (95% CI)**	**P**
Total N	202916	138093	11231		
Never	5612 (2.77%)	6575 (4.76%)	1078 (9.60%)	1 (Ref)	
Previous	4466 (2.20%)	5233 (3.79%)	1036 (9.22%)	1.39 (1.23-1.57)	6.49×10^{-8}
Current	192838 (95.03%)	126285 (91.45%)	9117 (81.17%)	0.94 (0.86-1.04)	0.218

Among former drinkers, 47.67% (5117/10735) explicitly indicated that they stopped drinking alcohol because they have poor health or have been advised not to. In examining the prospective association between frailty status and depression incidence, we included alcohol drinker status rather than alcohol intake frequency and found nearly unchanged results. Specifically, the risk was 1.61-times (HR=1.61, 95% CI=[1.55, 1.68], 4.12×10^{-122}) higher for pre-frailty and 3.28-times (HR=3.28, 95% CI=[3.06, 3.51], 2.91×10^{-243}) higher for frailty, compared with non-frail individuals. Regarding the prospective association between alcohol intake status and depression, we found a 1.39-times (HR=1.39, 95% CI=[1.23, 1.57], 6.49×10^{-8}) higher risk for former drinkers compared with never drinkers, while no significant difference was observed between never drinkers and current drinkers. All P values were two-sided, and no adjustments were made for multiple comparisons.

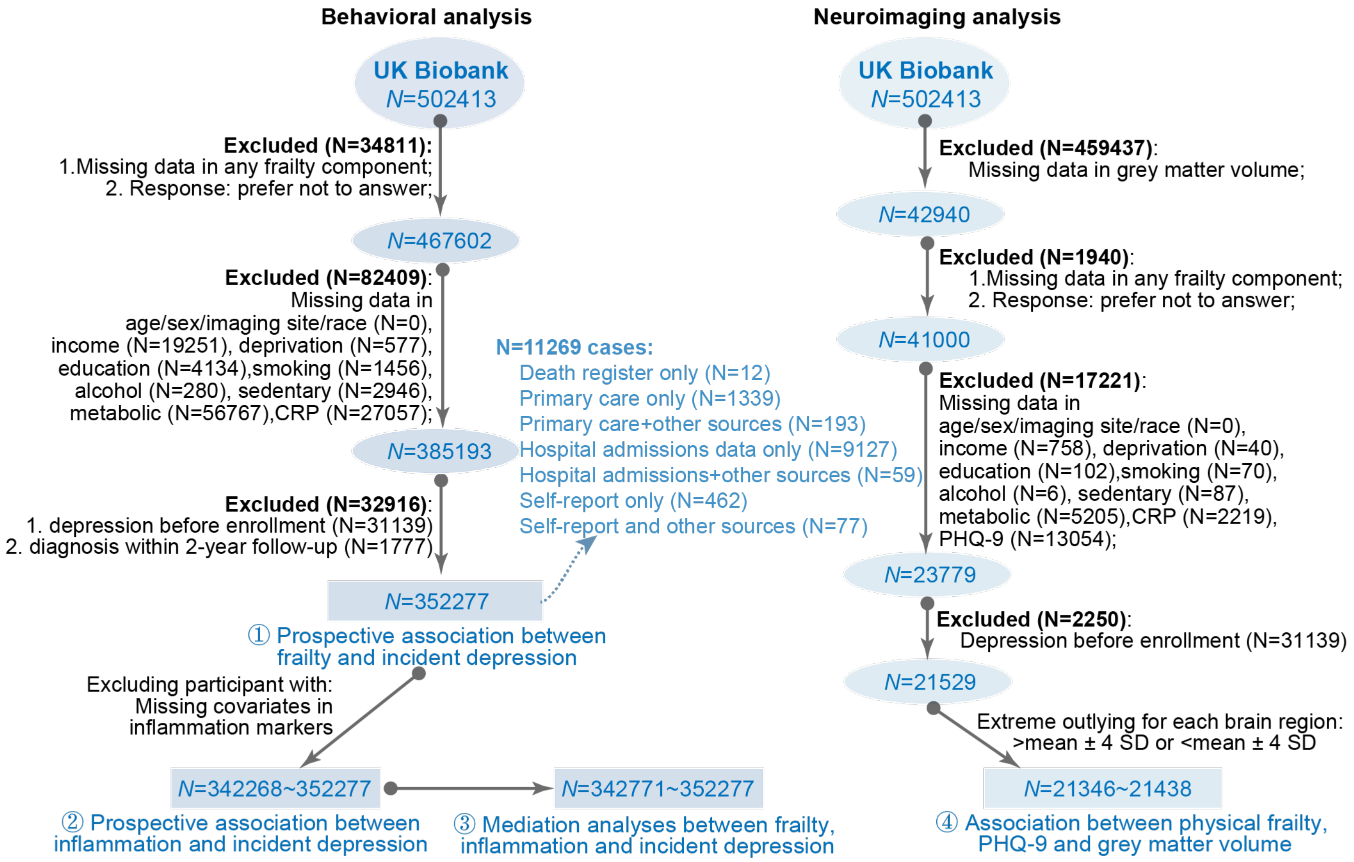

Supplementary Figure 7. Flowchart illustrating criteria for selection of samples for all analyses performed in the present study. We excluded participants who had missing data or responded “prefer not to answer” or “do not know” for any of the covariates and five indicators of frailty.

Supplementary table 15. Definition of physical frailty

Frailty indicators	UK Biobank question
Weight loss	Question: Compared with one year ago, has your weight changed?<br>· Yes - lost weight=1;<br>· No - weigh about the same=0;<br>· Yes - gained weight=0;<br>· Do not know=excluded;<br>· Prefer not to answer=excluded
Exhaustion	Question: Over the past two weeks, how often have you felt tired or had little energy?<br>· Not at all=0;<br>· Several days=0;<br>· More than half the days=1;<br>· Nearly every day=1;<br>· Do not know=excluded;<br>· Prefer not to answer=excluded
Slow walking speed	Question: How would you describe your usual walking pace?<br>· Slow pace=1;<br>· Steady average pace=0;<br>· Brisk pace=0;<br>· None of the above=excluded;<br>· Prefer not to answer=excluded
Weakness	Grip strength was assessed isometrically using a calibrated J00105 hydraulic hand dynamometer (Lafayette Instrument Company, IN, USA), separately in the left and right arms. The average of the right and left measurement was used here, or the available one when either measurement is missing[1].<br>Cut-offs used to define low grip strength:<br>· Males:<br>BMI≤24 & grip strength≤29;<br>24.1≤BMI≤28 & grip strength≤30;<br>BMI>28 & grip strength≤32<br>· Females:<br>BMI≤23 & grip strength≤17;<br>23.1≤BMI≤26 & grip strength≤17.3;<br>26.1≤BMI≤29 & grip strength≤18;<br>BMI>29 & grip strength≤21
Physical inactivity	Question: In the last 4 weeks did you spend any time doing the following?<br>· Walking for pleasure =0;<br>· Strenuous sports=0;<br>· Light DIY (*eg*: pruning, watering the lawn):<br>Frequency of once per week or less=1;<br>Frequency of more than once per week=0;<br>· Heavy DIY (*eg*: weeding, lawn mowing, carpentry, digging) =0;<br>· Other exercises (*eg*: swimming, cycling, keep fit, bowling) =0;<br>· None of the above=1;<br>· Prefer not to answer=excluded

Supplementary table 16. UK Biobank showcase variables used in all analyses in the study

Variable	Field ID	UK Biobank showcase link
**Demographic, socioeconomic, and lifestyle**		
Age	21003	https://biobank.ndph.ox.ac.uk/showcase/field.cgi?id=21003
Self-reported sex	31	https://biobank.ndph.ox.ac.uk/showcase/field.cgi?id=31
Assessment date	53	https://biobank.ndph.ox.ac.uk/showcase/field.cgi?id=53
Assessment center	54	https://biobank.ndph.ox.ac.uk/showcase/field.cgi?id=54
Race	21000	https://biobank.ndph.ox.ac.uk/showcase/field.cgi?id=21000
Material deprivation	189	https://biobank.ndph.ox.ac.uk/showcase/field.cgi?id=189
Education	6138	https://biobank.ndph.ox.ac.uk/showcase/field.cgi?id=6138
Family income	738	https://biobank.ndph.ox.ac.uk/showcase/field.cgi?id=738
Smoking status	20116	https://biobank.ndph.ox.ac.uk/showcase/field.cgi?id=20116
Alcohol intake	1558	https://biobank.ndph.ox.ac.uk/showcase/field.cgi?id=1558
Time spent watching TV	1070	https://biobank.ndph.ox.ac.uk/showcase/field.cgi?id=1070
Waist	48	https://biobank.ndph.ox.ac.uk/showcase/field.cgi?id=48
Body mass index	21001	https://biobank.ndph.ox.ac.uk/showcase/field.cgi?id=21001
Diabetes diagnosed by doctor	2443	https://biobank.ndph.ox.ac.uk/showcase/field.cgi?id=2443
Glucose	30740	https://biobank.ndph.ox.ac.uk/showcase/field.cgi?id=30740
Diastolic blood pressure	4079	https://biobank.ndph.ox.ac.uk/showcase/field.cgi?id=4079
Systolic blood pressure	4080	https://biobank.ndph.ox.ac.uk/showcase/field.cgi?id=4080
Vascular/heart problems diagnosed by doctor	6150	https://biobank.ndph.ox.ac.uk/showcase/field.cgi?id=6150
Triglycerides	30870	https://biobank.ndph.ox.ac.uk/showcase/field.cgi?id=30870
HDL cholesterol	30760	https://biobank.ndph.ox.ac.uk/showcase/field.cgi?id=30760
**Depression and physical frailty**		
Date F32 first reported (depressive episode)	130894	https://biobank.ndph.ox.ac.uk/showcase/field.cgi?id=130894
Source of report of F32 (depressive episode)	130895	https://biobank.ndph.ox.ac.uk/showcase/field.cgi?id=130895
Date F33 first reported (recurrent depressive disorder)	130896	https://biobank.ndph.ox.ac.uk/showcase/field.cgi?id=130896
Source of report of F33 (recurrent depressive disorder)	130897	https://biobank.ndph.ox.ac.uk/showcase/field.cgi?id=130897
Weight change compared with 1 year ago	2306	https://biobank.ndph.ox.ac.uk/showcase/field.cgi?id=2306
Frequency of tiredness / lethargy in last 2 weeks	2080	https://biobank.ndph.ox.ac.uk/showcase/field.cgi?id=2080
Usual walking pace	924	https://biobank.ndph.ox.ac.uk/showcase/field.cgi?id=924
Hand grip strength (left)	46	https://biobank.ndph.ox.ac.uk/showcase/field.cgi?id=46
Hand grip strength (right)	47	https://biobank.ndph.ox.ac.uk/showcase/field.cgi?id=47
Types of physical activity in last 4 weeks	6164	https://biobank.ndph.ox.ac.uk/showcase/field.cgi?id=6164
Frequency of light DIY in last 4 weeks	1011	https://biobank.ndph.ox.ac.uk/showcase/field.cgi?id=1011
Reason lost to follow-up	190	https://biobank.ndph.ox.ac.uk/showcase/field.cgi?id=190

Date lost to follow-up	191	https://biobank.ndph.ox.ac.uk/showcase/field.cgi?id=191
Recent feelings of inadequacy	20507	https://biobank.ndph.ox.ac.uk/showcase/field.cgi?id=20507
Recent trouble concentrating on things	20508	https://biobank.ndph.ox.ac.uk/showcase/field.cgi?id=20508
Recent feelings of depression	20510	https://biobank.ndph.ox.ac.uk/showcase/field.cgi?id=20510
Recent poor appetite or overeating	20511	https://biobank.ndph.ox.ac.uk/showcase/field.cgi?id=20511
Recent thoughts of suicide or self-harm	20513	https://biobank.ndph.ox.ac.uk/showcase/field.cgi?id=20513
Recent lack of interest or pleasure in doing things	20514	https://biobank.ndph.ox.ac.uk/showcase/field.cgi?id=20514
Trouble falling or staying asleep, or sleeping too much	20517	https://biobank.ndph.ox.ac.uk/showcase/field.cgi?id=20517
Recent changes in speed/amount of moving or speaking	20518	https://biobank.ndph.ox.ac.uk/showcase/field.cgi?id=20518
Recent feelings of tiredness or low energy	20519	https://biobank.ndph.ox.ac.uk/showcase/field.cgi?id=20519
**Inflammation markers**		
C-reactive protein	30710	https://biobank.ndph.ox.ac.uk/showcase/field.cgi?id=30710
White blood cell (leukocyte) count	30000	https://biobank.ndph.ox.ac.uk/showcase/field.cgi?id=30000
Platelet count	30080	https://biobank.ndph.ox.ac.uk/showcase/field.cgi?id=30080
Lymphocyte count	30120	https://biobank.ndph.ox.ac.uk/showcase/field.cgi?id=30120
Monocyte count	30130	https://biobank.ndph.ox.ac.uk/showcase/field.cgi?id=30130
Neutrophil count	30140	https://biobank.ndph.ox.ac.uk/showcase/field.cgi?id=30140
Lymphocyte percentage	30180	https://biobank.ndph.ox.ac.uk/showcase/field.cgi?id=30180
Monocyte percentage	30190	https://biobank.ndph.ox.ac.uk/showcase/field.cgi?id=30190
Neutrophil percentage	30200	https://biobank.ndph.ox.ac.uk/showcase/field.cgi?id=30200
**Neuroimaging markers**		
Head scaling factor	25000	https://biobank.ndph.ox.ac.uk/showcase/field.cgi?id=25000
Cortical volume	25782-25877	https://biobank.ndph.ox.ac.uk/showcase/label.cgi?id=1101
Subcortical volume	25011-25025	https://biobank.ndph.ox.ac.uk/showcase/label.cgi?id=1102

Supplementary methods information

Covariates

A total of 10 covariates were included in all analyses.

(1) Age was determined from the date of birth and the date of the baseline assessment. Participants were classified as middle-aged or older based on the cutoff of 65 years.

(2) Sex was self-reported.

(3) Race was self-reported and dichotomized as white and non-white (participants reporting 'prefer not to answer' or 'do not know' only account for a small proportion (<0.5%) of the entire population and were collapsed as non-white).

(4) Area-based socioeconomic status was calculated from residential postcodes using the Townsend scores with higher scores indicating fewer resources. The deprivation scores were further coded as tertiles for better interpretation.

(5) Educational attainment was self-reported and dichotomized as with and without university or college degree-level qualifications.

(6) Average total household income was self-reported and categorized as low (<£51,999), middle (£52,000–£100,000), and high (>£100,00) [2]. Since participants reporting "prefer not to answer" accounted for a large proportion of samples (9.43%), we created a separate 'Unknown' group for these individuals to maximize power.

(7) Self-reported smoking status including never, ever, and current smokers. We collapsed ever and current smokers as a single group.

(8) Participants reporting television watching time over four hours each day were classified as having sedentary behavior[3].

(9) Self-reported alcohol intake frequency ranged from 'never' to 'daily or almost daily' and was coded as an ordinary variable.

(10) Metabolic syndrome was defined as the occurrence of any three or more of the following components, including central obesity, high glycaemia/diabetes, hypertension, low HDL, and high triglycerides[4]. Central obesity was defined as a waist circumference >88 cm for females and >102 cm for males. High glycaemia/diabetes was defined as fasting glucose ≥5.6 mmol/L or self-report of a physician's diagnosis of diabetes. Hypertension was defined as a systolic blood pressure ≥130 mmHg and/or a diastolic blood pressure ≥85 mmHg or self-report of a physician's diagnosis of hypertension. High triglycerides were defined as ≥1.7 mmol/L. Low HDL-cholesterol was <1.3 mmol/L in females and <1.0 mmol/L in males[5].

PHQ-9 assessment

Depressive symptoms were measured using the nine-item version of the Patient Health Questionnaire (PHQ-9)[6], which assesses how often over the past 2 weeks an individual experienced each of the 9 depressive symptoms:

(1) Recent feelings of inadequacy.
(2) Recent trouble concentrating on things.
(3) Recent feelings of depression.
(4) Recent poor appetite or overeating.
(5) Recent thoughts of suicide or self-harm.
(6) Recent lack of interest or pleasure in doing things.
(7) Trouble falling or staying asleep or sleeping too much.
(8) Recent changes in speed/amount of moving or speaking.
(9) Recent feelings of tiredness or low energy.

Total scores were computed by summing individual item responses. Notably, the last item (recent feelings of tiredness or low energy) was excluded from the PHQ-9 calculation, due to its overlap with the exhaustion component of physical frailty.

Supplementary References

1. Petermann-Rocha F, Hanlon P, Gray SR, et al. Comparison of two different frailty measurements and risk of hospitalisation or death from COVID-19: findings from UK Biobank. *BMC Med* 2020; **18**(1): 355.
2. Jiang R, Noble S, Sui J, et al. Associations of physical frailty with health outcomes and brain structure in 483 033 middle-aged and older adults: a population-based study from the UK Biobank. *Lancet Digit Health* 2023; **5**(6): e350-e9.
3. Foster HME, Celis-Morales CA, Nicholl BI, et al. The effect of socioeconomic deprivation on the association between an extended measurement of unhealthy lifestyle factors and health outcomes: a prospective analysis of the UK Biobank cohort. *The Lancet Public Health* 2018; **3**(12): e576-e85.
4. Huang PL. A comprehensive definition for metabolic syndrome. *Dis Model Mech* 2009; **2**(5-6): 231-7.
5. Petermann-Rocha F, Gray SR, Forrest E, et al. Associations of muscle mass and grip strength with severe NAFLD: A prospective study of 333,295 UK Biobank participants. *J Hepatol* 2022; **76**(5): 1021-9.
6. Gilbody S, Richards D, Brealey S, Hewitt C. Screening for depression in medical settings with the Patient Health Questionnaire (PHQ): a diagnostic meta-analysis. *J Gen Intern Med* 2007; **22**(11): 1596-602.
7. Daviet R, Aydogan G, Jagannathan K, et al. Associations between alcohol consumption and gray and white matter volumes in the UK Biobank. *Nature Communications* 2022; **13**(1): 1-11.
8. Alfaro-Almagro F, Jenkinson M, Bangerter NK, et al. Image processing and Quality Control for the first 10,000 brain imaging datasets from UK Biobank. *Neuroimage* 2018; **166**: 400-24.
9. Miller KL, Alfaro-Almagro F, Bangerter NK, et al. Multimodal population brain imaging in the UK Biobank prospective epidemiological study. *Nat Neurosci* 2016; **19**(11): 1523-36.
10. Davies NM, Holmes MV, Davey Smith G. Reading Mendelian randomisation studies: a guide, glossary, and checklist for clinicians. *BMJ* 2018; **362**: k601.
11. Ye Y, Noche RB, Szejko N, et al. A genome-wide association study of frailty identifies significant genetic correlation with neuropsychiatric, cardiovascular, and inflammation pathways. *Geroscience* 2023; **45**(4): 2511-23.
12. Wray NR, Ripke S, Mattheisen M, et al. Genome-wide association analyses identify 44 risk variants and refine the genetic architecture of major depression. *Nat Genet* 2018; **50**(5): 668-81.
13. Hemani G, Zheng J, Elsworth B, et al. The MR-Base platform supports systematic causal inference across the human phenome. *eLife* 2018; **7**.
14. Petermann-Rocha F, Lyall DM, Gray SR, et al. Associations between physical frailty and dementia incidence: a prospective study from UK Biobank. *Lancet Health Longev* 2020; **1**(2): E58-E68.
