# Supplementary material for: The brain structure, inflammatory, and genetic mechanisms mediate the association between physical frailty and depression
Source: Nat Commun. 2024 May 23;15:4411. doi: 10.1038/s41467-024-48827-8 (PMC11116547; doi:10.1038/s41467-024-48827-8)
Supplement: Supplementary file 3 — Reporting Summary [file 41467_2024_48827_MOESM3_ESM.pdf]

nature portfolio

Corresponding author(s): Rongtao Jiang

Last updated by author(s): Apr 20, 2024

Reporting Summary

Nature Portfolio wishes to improve the reproducibility of the work that we publish. This form provides structure for consistency and transparency in reporting. For further information on Nature Portfolio policies, see our Editorial Policies and the Editorial Policy Checklist.

Statistics

For all statistical analyses, confirm that the following items are present in the figure legend, table legend, main text, or Methods section.

n/a	Confirmed	
☐	☒	The exact sample size (*n*) for each experimental group/condition, given as a discrete number and unit of measurement
☐	☒	A statement on whether measurements were taken from distinct samples or whether the same sample was measured repeatedly
☐	☒	The statistical test(s) used AND whether they are one- or two-sided<br>*Only common tests should be described solely by name; describe more complex techniques in the Methods section.*
☐	☒	A description of all covariates tested
☐	☒	A description of any assumptions or corrections, such as tests of normality and adjustment for multiple comparisons
☐	☒	A full description of the statistical parameters including central tendency (e.g. means) or other basic estimates (e.g. regression coefficient) AND variation (e.g. standard deviation) or associated estimates of uncertainty (e.g. confidence intervals)
☐	☒	For null hypothesis testing, the test statistic (e.g. *F*, *t*, *r*) with confidence intervals, effect sizes, degrees of freedom and *P* value noted<br>*Give P values as exact values whenever suitable.*
☒	☐	For Bayesian analysis, information on the choice of priors and Markov chain Monte Carlo settings
☒	☐	For hierarchical and complex designs, identification of the appropriate level for tests and full reporting of outcomes
☐	☒	Estimates of effect sizes (e.g. Cohen's *d*, Pearson's *r*), indicating how they were calculated

Our web collection on statistics for biologists contains articles on many of the points above.

Software and code

Policy information about availability of computer code

Data collection	No software was involved in data collection (data used is all directly available from UK Biobank, as described in the paper)
Data analysis	R version 4.2.2 packages:<br>survival 3.5-0 was used to perform Cox proportional hazard regression model in studying the association between frailty and depression incidence;<br>rms 6.4-1 was used to perform nonlinear association analysis;<br>mediation 4.5.0 was used to perform mediation analysis;<br>lmerTest 3.1-3 was used to investigate the association of physical frailty and depression symptoms with inflammatory markers and regional grey matter volumes;<br>MatchIt 4.5.3 was used to perform the propensity score matching analysis;<br>TwoSampleMR 0.5.8 was used to perform Mendelian randomization study.

For manuscripts utilizing custom algorithms or software that are central to the research but not yet described in published literature, software must be made available to editors and reviewers. We strongly encourage code deposition in a community repository (e.g. GitHub). See the Nature Portfolio guidelines for submitting code & software for further information.

Data

Policy information about availability of data

All manuscripts must include a data availability statement. This statement should provide the following information, where applicable:

- Accession codes, unique identifiers, or web links for publicly available datasets
- A description of any restrictions on data availability
- For clinical datasets or third party data, please ensure that the statement adheres to our policy

The UK Biobank data are available via their standard data access procedure at https://www.ukbiobank.ac.uk/ with access fees. Researchers can apply for access to the UK Biobank data via the Access Management System (AMS) (https://www.ukbiobank.ac.uk/enable-your-research/apply-for-access).

Research involving human participants, their data, or biological material

Policy information about studies with human participants or human data. See also policy information about sex, gender (identity/presentation), and sexual orientation and race, ethnicity and racism.

Reporting on sex and gender

Our study took sex into considerations and our findings could apply to both male and female. We also repeated our analyses stratified by sex. Sex (Field ID 31) in the UK Biobank was determined based on self-reporting data via questionnaire. All 352277 participants gave written informed consent for sharing of individual-level data.

Reporting on race, ethnicity, or other socially relevant groupings

A total of 10 covariates were included in all analyses.

(1) Age was determined from the date of birth and the date of the baseline assessment. Participants were classified as middle-aged or older based on the cutoff of 65 years.

(2) Sex was self-reported.

(3) Race was self-reported and dichotomized as white and non-white (participants reporting 'prefer not to answer' or 'do not know' only account for a small proportion (<0.5%) of the entire population and were collapsed as non-white).

(4) Area-based socioeconomic status was calculated from residential postcodes using the Townsend scores with higher scores indicating fewer resources. The deprivation scores were further coded as tertiles for better interpretation.

(5) Educational attainment was self-reported and dichotomized as with and without university or college degree-level qualifications.

(6) Average total household income was self-reported and categorized as low (<£51,999), middle (£52,000–£100,000), and high (>£100,00). Since participants reporting "prefer not to answer" accounted for a large proportion of samples (9.43%), we created a separate 'Unknown' group for these individuals to maximize power.

(7) Self-reported smoking status including never, ever, and current smokers. We collapsed ever and current smokers as a single group.

(8) Participants reporting television watching time over four hours each day were classified as having sedentary behavior.

(9) Self-reported alcohol intake frequency ranged from 'never' to 'daily or almost daily' and was coded as an ordinary variable.

(10) Metabolic syndrome was defined as the occurrence of any three or more of the following components, including central obesity, high glycaemia/diabetes, hypertension, low HDL, and high triglycerides. Central obesity was defined as a waist circumference >88 cm for females and >102 cm for males. High glycaemia/diabetes was defined as fasting glucose ≥5.6 mmol/L or self-report of a physician's diagnosis of diabetes. Hypertension was defined as a systolic blood pressure ≥130 mmHg and/or a diastolic blood pressure ≥85 mmHg or self-report of a physician's diagnosis of hypertension. High triglycerides were defined as ≥1.7 mmol/L. Low HDL-cholesterol was <1.3 mmol/L in females and <1.0 mmol/L in males.

Population characteristics

A total of 352,277 participants were included in the final analyses, of whom 11,241 met the criteria for frailty, 138,111 for pre-frailty, and 202,925 for non-frailty. Participants were 51.77% female, 95.15% White, and had a mean age of 56.48 years. Baseline characteristics by frailty status are detailed in Supplement. During a median follow-up of 12.25 years (IQR 11.52 to 12.94 years), 11,269 depression cases were documented. We calculated descriptive statistics as mean (SD) for continuous variables and number (percentage) for categorical variables.

Recruitment

The UK Biobank is a population-based cohort study of over 500,000 participants aged 37-73 years. Between 2006 and 2010, participants attended one of 22 assessment centers, where they completed touchscreen and nurse-led questionnaires, had physical measurements taken, and provided biological samples. Since 2014, a subsample of participants was invited back for imaging assessment. The database is linked to national health datasets, including primary care, hospital inpatient, death, and cancer registration data.

Ethics oversight

The UK Biobank study was approved by the North West Multicenter Research Ethics Committee (No.11/NW/0382), and written informed consent was obtained from all participants. This research was conducted using the UK Biobank resources (application number 42009).

Note that full information on the approval of the study protocol must also be provided in the manuscript.

Field-specific reporting

[illegible]

For a reference copy of the document with all sections, see nature.com/documents/nr-reporting-summary-flat.pdf

Life sciences study design

All studies must disclose on these points even when the disclosure is negative.

Sample size	No statistical methods were used to predetermine sample sizes. All currently available sample in the UK Biobank were included. A total of 352,277 participants were included in the final analyses. Flowchart illustrating criteria for selection of samples for all analyses performed in the present study can be found in Supplement. We excluded participants who had missing data or responded "prefer not to answer" or "do not know" for any of the covariates and five indicators of frailty.
Data exclusions	We excluded participants who had missing data or responded "prefer not to answer" or "do not know" for any of the covariates and five frailty indicators; To minimize possible reverse causality, we also performed a 2-year landmark analysis by excluding participants who experienced events within the first 2 years of follow-up; Participants with missing data for any covariates were also excluded. Flowchart illustrating criteria for selection of samples for all analyses performed in the present study can be found in Supplement.
Replication	To validate the results, we conducted a propensity score matching analysis using the "MatchIt" package in R. Participants with frailty were matched on all 10 covariates to a single participant with non-frailty (1:1 ratio nearest-neighbor matching without replacement). Based on the matched data, we repeated our analyses.
Randomization	Covariates including age, sex, race, alcohol intake, smoking status, sedentary behavior, education level, material deprivation, family income, and metabolic syndrome were adjusted in our study.
Blinding	Not applicable.

Reporting for specific materials, systems and methods

We require information from authors about some types of materials, experimental systems and methods used in many studies. Here, indicate whether each material, system or method listed is relevant to your study. If you are not sure if a list item applies to your research, read the appropriate section before selecting a response.

Materials & experimental systems

n/a	Involved in the study	
☒	☐	Antibodies
☒	☐	Eukaryotic cell lines
☒	☐	Palaeontology and archaeology
☒	☐	Animals and other organisms
☒	☐	Clinical data
☒	☐	Dual use research of concern
☒	☐	Plants

Methods

n/a	Involved in the study	
☒	☐	ChIP-seq
☒	☐	Flow cytometry
☐	☒	MRI-based neuroimaging

Plants

Seed stocks	*Report on the source of all seed stocks or other plant material used. If applicable, state the seed stock centre and catalogue number. If plant specimens were collected from the field, describe the collection location, date and sampling procedures.*
Novel plant genotypes	*Describe the methods by which all novel plant genotypes were produced. This includes those generated by transgenic approaches, gene editing, chemical/radiation-based mutagenesis and hybridization. For transgenic lines, describe the transformation method, the number of independent lines analyzed and the generation upon which experiments were performed. For gene-edited lines, describe the editor used, the endogenous sequence targeted for editing, the targeting guide RNA sequence (if applicable) and how the editor was applied.*
Authentication	*Describe any authentication procedures for each seed stock used or novel genotype generated. Describe any experiments used to assess the effect of a mutation and, where applicable, how potential secondary effects (e.g. second site T-DNA insertions, mosiacism, off-target gene editing) were examined.*

Magnetic resonance imaging

Experimental design

Design type	Structural MRI.
Design specifications	T1-weighted structural image was acquired using straight sagittal orientation for 5 minutes.
Behavioral performance measures	Not applicable.

Acquisition

Imaging type(s)	T1-weighted structural imaging
Field strength	3.0 T
Sequence & imaging parameters	All brain MRI data were acquired on a 3T Siemens Skyra scanner using a standard 32-channel head coil. Of relevance to this study, T1-weighted MPRAGE were obtained in sagittal orientation using the following parameters: resolution: 1×1×1 mm, field-of-view (FOV): 208×256×256 matrix, duration: 5 minutes; T2-weighted FLAIR volumes were acquired in sagittal orientation using the following parameters: resolution: 1.05×1×1 mm, FOV: 192×256×256 matrix, duration: 6 minutes.
Area of acquisition	Whole brain.

Diffusion MRI ☐ Used ☒ Not used

Preprocessing

Preprocessing software	Brain MRI data were acquired and processed by the UK Biobank team and made available to approved researchers as image-derived phenotypes (IDP).<br>The tissue-type segmentation was applied using FAST (FMRIB's Automated Segmentation Tool), and subcortical structures were modeled using FIRST (FMRIB's Integrated Registration and Segmentation Tool). The 110 brain regions include 96 cortical and 14 subcortical regions based on Harvard-Oxford atlas (https://fsl.fmrib.ox.ac.uk/fsl/fslwiki/Atlases). GMV segmentation based on FAST and FIRST is one of the most widely used methods to delineate gray matter structures from MR images. Details regarding data acquisition protocols and preprocessing can be found at https://biobank.ctsu.ox.ac.uk/crystal/crystal/docs/brain_mri.pdf and elsewhere.
Normalization	see above.
Normalization template	see above
Noise and artifact removal	see above
Volume censoring	see above

Statistical modeling & inference

Model type and settings	Linear-mixed effect models were applied to investigate the association of physical frailty and depression symptoms with inflammatory markers and regional GMVs. Within the same analytical framework, physical frailty (or PHQ-9) was fitted as a fixed effect, UK Biobank assessment center as a random effect, and each of the nine inflammatory markers (or regional GMVs) was set as the dependent variable in separate models. The same set of covariates as listed above were used here.
Effect(s) tested	We extracted the standardized beta coefficients and converted them to Cohen's d according to a previous study.

Specify type of analysis: ☐ Whole brain ☒ ROI-based ☐ Both

Anatomical location(s)	The 110 brain regions include 96 cortical and 14 subcortical regions based on Harvard-Oxford atlas (https://fsl.fmrib.ox.ac.uk/fsl/fslwiki/Atlases).
Statistic type for inference<br>(See Eklund et al. 2016)	Not applicable
Correction	Brain association analyses were corrected using the Benjamini-Hochberg false discovery rate (FDR) method due to the many brain regions.

Models & analysis

n/a	Involved in the study
☒	☐ Functional and/or effective connectivity
☒	☐ Graph analysis
☒	☐ Multivariate modeling or predictive analysis
